# PASTA: An Efficient Proactive Adaptation Approach Based on Statistical Model Checking for Self-Adaptive Systems

**DOI:** 10.1007/978-3-030-71500-7_15

**Published:** 2021-02-24

**Authors:** Yong-Jun Shin, Eunho Cho, Doo-Hwan Bae

**Affiliations:** 8 Universidad Autónoma de Madrid, Madrid, Spain; 9 University of Twente, Enschede, The Netherlands; Korea Advanced Institute of Science and Technology (KAIST), Deajeon, Republic of Korea

**Keywords:** Self-adaptive system, Proactive adaptation, Statistical model checking, Environmental uncertainty

## Abstract

Proactive adaptation, in which the adaptation for a system’s reliable goal achievement is performed by predicting changes in the environment, is considered as an effective alternative to reactive adaptation, in which adaptation is performed after observing changes. When predicting the environmental changes, the prediction may be uncertain, so it is necessary to verify and confirm an adaptation’s consequences before execution. To resolve the uncertainty, probabilistic model checking (PMC) has been utilized for verification of adaptation tactics’ effects on the goal of a self-adaptive system (SAS). However, PMC-based approaches have limitations on the state-explosion problem of complex SAS model verification and the modeling languages supported by the model checkers. In this paper, to overcome the limitations of the PMC-based approaches, we propose an efficient Proactive Adaptation approach based on STAtistical model checking (PASTA). Our approach allows SASs to mitigate the uncertainty of the future environment, faster than the PMC-based approach, by producing statistically sufficient samples for verification of adaptation tactics based on statistical model checking (SMC) algorithms. We provide algorithmic processes, a reference architecture, and an open-source implementation skeleton of PASTA for engineers to apply it for SAS development. We evaluate PASTA on two SASs using actual data and show that PASTA is efficient comparing to the PMC-based approach. We also provide a comparative analysis of the advantages and disadvantages of PMC- and SMC-based proactive adaptation to guide engineers’ decision-making for SAS development.

### 1 Introduction

As the complexity of an environment that affects a system's goal achievement increases, analyzing the environment becomes important for reliable goal achievement. The environment, such as user traffic and outdoor temperatures, can change over time [15,29]. Full anticipation of environmental changes at the system design time is challenging and often impossible [6,9]. Systems are required to be self-adaptive so that they change their behaviors and structures according to the environmental changes at runtime. To realize this, numerous design approaches [11,13,14,16] have been proposed based on the MAPE feedback loop [18]. These adaptation processes involve the continual monitoring and analysis of the environment as well as the planning and execution of the adaptation.

For most existing approaches, adaptation has been reactively triggered by system failures or changes in the environment [12,31,33]. Other adaptation approaches, known as proactive or predictive adaptation, have emerged, which have proven to be more effective than reactive adaptations in a changing environment by predicting changes in advance [2,24,26]; however, the prediction of environmental changes is uncertain, so the uncertainty affects the consequences of proactive adaptation. To resolve the uncertainty, probabilistic model checking (PMC) was utilized in some studies for the verification of adaptation tactics and their effects on the system's adaptation goal [5,26,27,28].

PMC-based approaches are a major method used for proactive adaptation; however, PMC may be not appropriate for the verification of large and complex self-adaptive system (SAS) models due to the state explosion problem. PMC requires a high verification cost in time and memory to fully examine the given probabilistic models, so the verification of complex SAS models and adaptation tactics may fail due to time and memory constraints. In addition, modeling languages supported by probabilistic model checkers must be used for the modeling of the SAS and the environment. Engineers must be familiar with modeling languages, such as Markov chains, Markov decision processes, or automata, that model checkers can interpret [21]. To overcome the limitations, we propose an efficient proactive adaptation approach based on statistical model checking (SMC) that consumes a smaller verification resource than PMC and only requires simulation results of system models without limiting languages.

Our Proactive Adaptation approach based on STAtistical model checking (PASTA) offers the following contributions:

- We propose a proactive adaptation approach utilizing SMC to eliminate the uncertainty of the future environment faster than PMC for the verification of adaptation tactics.
- We provide algorithmic processes, a reference architecture, and an open-source implementation skeleton of PASTA for developers who will apply PASTA to SAS development.
- Based on evaluations using actual data, we also provide a comparative analysis of the advantages and disadvantages of PMC- and SMC-based proactive adaptation to guide engineers' decision making.

The remainder of this paper is organized as follows. Section 2 introduces related work of proactive adaptation. Section 3 provides the background knowledge of SMC. Section 4 presents an illustrative example. Section 5 introduces our PASTA approach. Section 6 evaluates PASTA based on two SASs with actual data. Section 7 reveals the threats and validity of our work. Section 8 concludes the paper.

### 2 Related Work: Proactive Adaptation

Numerous studies on proactive or predictive adaptation have been conducted to address issues related to changing environments [3,20,24,25]. As opposed to reacting to changes in the environment or system, predicting and responding to the predicted situations could be more difficult but more effective in preventing system failures and meeting requirements. Many case studies on proactive adaptation have been conducted, and it has been demonstrated that proactive adaptation outperforms reactive adaptation in terms of the system's adaptation goal [2,10,20]. For proactive adaptation, the prediction of the future environment is uncertain, so approaches utilizing probabilistic model checking (PMC), which verifies the property satisfaction of probabilistic model, have been proposed to provide verified and trustworthy proactive adaptation results [5,26,27,28]. The main process of PMC-based proactive adaptation is illustrated in Fig. 1. Core of the process are the formal modeling of the future environment, system, and adaptation tactics, and the verification of the models to identify an optimal adaptation tactic for adaptation goal achievement. However, PMC is not appropriate for the verification of large and complex models due to its state explosion problem. It requires exhaustively examining all possible states of SAS models to verify adaptation tactics. It also requires engineers to develop SAS models written in modeling languages that model checkers can support. To tackle the limitations, as an alternative to PMC-based approaches, which have been the major trend of proactive adaptation, in this paper, we propose a statistical model checking (SMC)-based proactive adaptation approach [19,23,34].

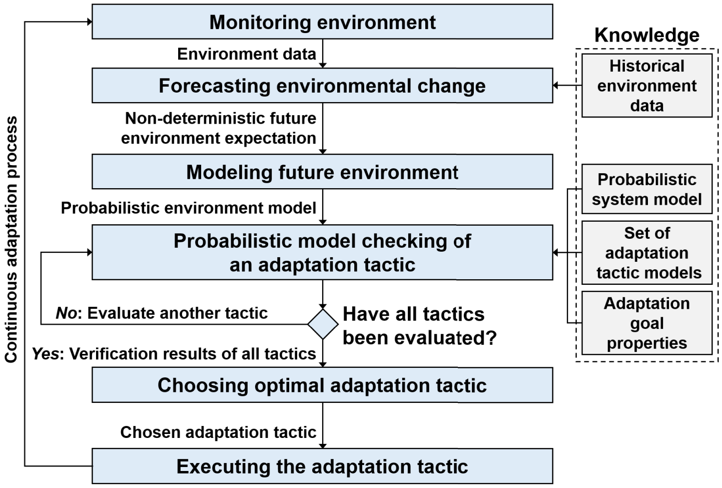

**Fig. 1.** PMC-based proactive adaptation process

### 3 Background: Statistical Model Checking (SMC)

We have utilized statistical model checking (SMC) to verify adaptation tactics at runtime under an uncertain environment. SMC is an efficient technique for verifying a stochastic model [22,23]. Although PMC exhaustively examines the model, SMC simulates the model to obtain samples and provides statistical evidence of the satisfaction or violation of the given property using hypothesis testing for the samples. In fact, SMC requires only a set of simulation results, so it can be applied to an executable black-box model or to only a set of simulation results. The fact that the verification results depend on the quality of the model is the same as PMC. However, as it is a simulation-based approach, it is known to be an efficient alternative to PMC in terms of time and memory, performing verification with a certain confidence [1,19]. In this regard, SMC can be used effectively for the runtime verification of SAS adaptation tactics with uncertain environments. The following examples of SMC algorithms are widely used:

– ***Simple Monte Carlo Simulation (SMCS).*** This is the simplest and most intuitive SMC algorithm [1,4]. It estimates the quantitative satisfaction of a property according to the ratio of samples that satisfy the property in the overall samples. It requires a fixed number of samples from the user.
– ***Single Sampling Plan (SSP).*** The SSP [34] tests a hypothesis $H : p \geq \theta$ with fixed-size samples, where $p$ is the probability that a system meets a given property and $\theta$ is the verification threshold of $p$. The user provides two error bounds $\alpha$ $(0 \leq \alpha \leq 1)$ and $\beta$ $(0 \leq \beta \leq 1)$ of false negatives and false positives, respectively. Within the given error bounds, the SSP estimates $p$ to accept or to reject $H$. The detailed algorithm can be found in [19,23,34].
– ***Sequential Probability Ratio Test (SPRT).*** Similar to the SSP, the SPRT [32] tests a hypothesis $H$ within the given error bounds, but the number of samples is determined automatically. It simulates the target system to obtain a sample, and iterates the simulations to generate sufficient samples until it can accept or reject $H$ within a given error bound. The detailed algorithm can be found in [19,23,34].

For the PASTA approach, an SMC algorithm is selected and used to obtain statistical evidence of an adaptation tactic's performance in a future environment to evaluate possible tactics and to identify the optimal tactic at runtime.

### 4 Illustrative Example

We illustrate PASTA using an adaptive air condition control system as an example. The system monitors indoor and outdoor air conditions, including temperature and humidity, and adaptively controls the indoor condition for a given target condition. Planning an adaptive air condition control with an immediate reaction to the monitored indoor condition can aid the system in achieving its goal; however, the indoor air conditions may change over time due to the influence of the outdoor air conditions, as shown in Fig. 2. If the adaptation plan is made without taking the environmental change into account, the adaptation consequences may differ from the expectations, and thus there could have been a better adaptation tactic that was not chosen. The air condition control system developed by the PASTA approach forecasts future air condition changes and selects an optimal adaptation tactic whose adaptation consequences are verified by SMC at runtime. Throughout this paper, we will describe our approach using this example.

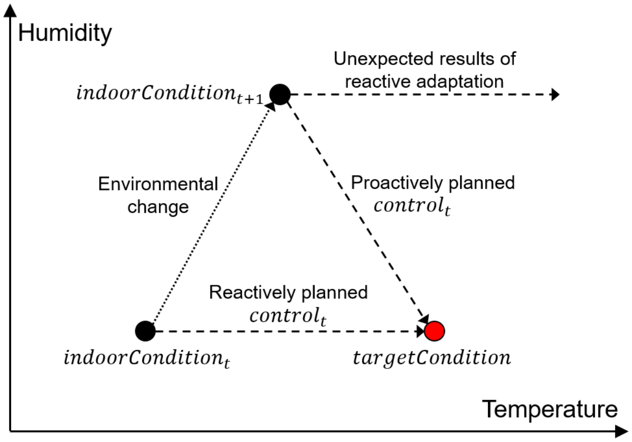

**Fig. 2.** Adaptive air condition control system

## 5 Proactive Adaptation Based on Statistical Model Checking

### 5.1 PASTA overview

We propose the PASTA approach, which is a proactive adaptation, using SMC. Fig. 3 presents the overall adaptation process. The aim of the approach is to provide efficient proactive adaptation based on the prediction of environmental changes and the verification of the adaptation tactics of the SAS. (Step 1) Initially, PASTA continuously monitors the environment to capture its change at runtime. (Step 2) It analyzes the monitored (historical) environment data and forecasts future environmental changes based on its forecasting algorithm. The prediction or expectation of the future environment is in the form of non-deterministic possibility, such as the probability density function of future environmental conditions. (Step 3) Based on the prediction, a sample of the possible future environment is made and given to the simulation engine as a simulation environment. (Step 4) In the given environment, an adaptation tactic is applied to the system model and simulated to make a sample evaluation of the tactic's performance. The simulations are repeated until the system obtains the statistically sufficient number of samples for the verification of the tactic's performance for the adaptation goal in the expected future environmental change. (Step 5) Based on the accumulated samples, the performance of an adaptation tactic is verified. All adaptation tactics are evaluated repeatedly in the same manner, and the SAS statistically guarantees the effects of its adaptation tactics. (Step 6 and 7) When all possible adaptation tactics have been evaluated, an optimal adaptation tactic is chosen and executed. This adaptation process is continuously repeated to respond to continuous environmental changes. We describe the PASTA approach in detail based on this adaptation process in the subsequent sections.

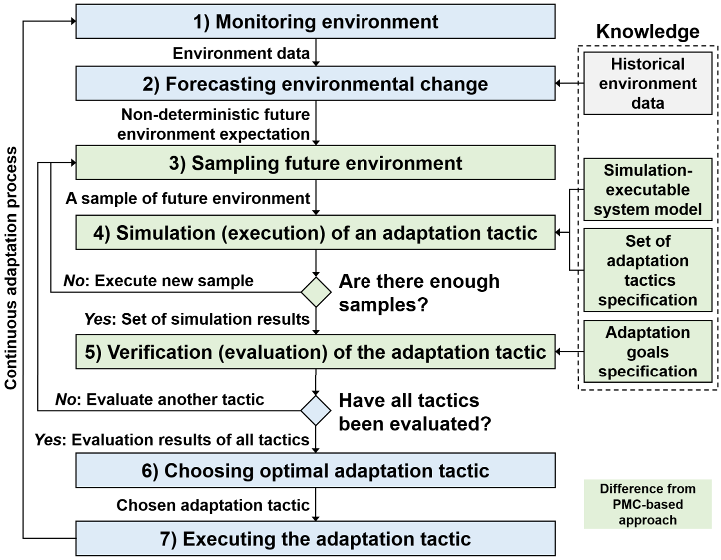

**Fig. 3.** Overall PASTA process

### 5.2 Knowledge

***Principle.*** The PASTA approach requires an SAS to accumulate the monitored environment data. The accumulated historical environment data is analyzed to predict environmental changes. Furthermore, the system has its current system model that is an abstraction of the system behavior executable by a simulator. The model in PASTA is user-specific, and although the modeling language and system information to be modeled are selected by the engineer, the only requirement is that the model is executable to generate simulation logs. The system model also contains a finite set (space) of possible adaptation tactics that will be verified. An adaptation tactic is a specification of an adaptation that can be applied to the SAS and its model, such as a set of configurations. The adaptation goal is also specified in the knowledge. Thus, the optimal tactic for the adaptation goals will be selected and executed.

***Example.*** The environmental factors of interest in the adaptive air condition control system are the indoor/outdoor temperature and humidity; therefore, the monitored environment data at a specific time include values of four factors. The simulation models imitate the changes of the indoor temperature and humidity affected by outdoor conditions and the air condition control system's control values. The system's possible adaptation tactics are defined by the system capabilities of each temperature and humidity control capability. For example, the system can increase or decrease the temperature and humidity in $0.1°C$ and 0.1% increments up to $5°C$ and 5%, respectively, in a discrete simulation time unit. The tactic space is a Cartesian product of the possible temperature and humidity controls. The adaptation goal is to manipulate the indoor temperature and humidity to the user's desired conditions.

### 5.3 Monitoring Environmental Changes

***Principle.*** (Step 1) The system constantly monitors the environment. The environment is measured as the values of the environmental conditions observable by the sensors. The current environmental data are added to the environment database. The current state of the system is also monitored, and the system model is kept up to date.

***Example.*** The air condition control system constantly monitors the indoor/outdoor temperature and humidity. It accumulates the environment data in its environment database.

### 5.4 Forecasting Future Environmental Change

***Principle.*** (Step 2) PASTA forecasts future environmental changes based on the accumulated historical environment data using a data analysis or forecasting techniques. As the given historical environmental data consist of time-series data, a time-series analysis and forecasting methods, such as random walk [30], errortrend-seasonal [17], autoregressive integrated moving average model [7], or any machine-learning techniques, can be applied, and the choice of the forecasting methods depends on domain engineers. What is important here is that the predictions of future environmental changes based on historical data are uncertain, so the results of the forecasting are non-deterministic expectations, such as the probability density function of future environmental conditions. This uncertainty will be resolved by SMC.

***Example.*** The system predicts the outdoor temperature and humidity changes, which exhibit distinct repetitive patterns (seasonality) at 24-hour intervals. As the environmental data of this system exhibit distinct seasonality, they can be predicted naively with a random walk model using seasonal differencing [17]. Based on the historical temperature data and the forecasting algorithm, the temperature change from the present to a few hours later can be predicted using the probability density function. For example, if the current temperature at 2

p.m. is $24°C$, the temperature at 3 p.m. can be expected to change according to the uniform distribution between $24°C$ and $30°C$.

#### 5.5 Planning Adaptation Using SMC

**Algorithm 1:** PASTA adaptation planning

```
Input  : envPrediction, sysModel, tacticSpace, goalProp
Output: optimalTactic
Procedure
    evaluationSheet = [];
    foreach tactic in tacticSpace do
        simulationResultList = [];
        while !samplesSufficient() do
            envSample = makeSample(envPrediction);
            simResult = simulate(envSample, sysModel, tactic);
            addElement(simulationResultList, simResult);
        end
        evaluationResult = verify(simulationResultList, goalProp);
        addElement(evaluationSheet, (tactic, evaluationResult));
    end
    optimalTactic = getOptimalTactic(evaluationSheet);
end
```

***Principle.*** The adaptation planning of the PASTA approach involves searching for the optimal tactic among possible adaptation tactics using SMC, as shown in Algorithm 1. Evaluating an adaptation tactic using SMC consists of three steps: sampling environmental changes, simulating adaptation tactics, and verifying the simulation results. (Step 3) The forecasting result is non-deterministic, so the sample generator produces a deterministic sample of possible future environmental conditions based on the forecasting result. SMC eliminates the uncertainty of the nondeterministic future environment by producing statistically sufficient samples, while PMC probabilistically verifies a stochastic model. The number of samples is determined depending on the SMC algorithms, as explained in the background section. (Step 4) The simulator takes the sample environment, the system model, and an adaptation tactic as inputs. It applies the given tactic to the system model, simulates the system in the sample of the future environment, and returns a simulation result logs that represents the effects of the adaptation tactic in the future environment. (Step 5) The verifier receives the numerous simulation results and evaluates the tactic's performance for the adaptation goal represented as a verification property. This process is performed for all adaptation tactics, and (Step 6) the optimal tactic is selected based on all evaluation (verification) results. Therefore, the planning time required for an adaptation depends on the number of tactics, the number of required samples, and the time for a single simulation of the model.

***Example.*** Based on the predicted range of the temperature change at 3 p.m. ($24°C \sim 30°C$), the samples of the future outdoor temperature (for example, $25°C$, $27°C$, and $29°C$) are randomly selected by an SMC algorithm. The system model and an adaptation tactic (for example, lower the indoor temperature by $3°C$) under the current evaluation are simulated with the sample environments, respectively. Based on the simulation results, the verifier evaluates the adaptation results of the indoor temperature control. In this example, the average distance between the target condition and the current condition is used as a verification property representing an adaptation goal, but the maximum distance indicating the worst case, the presence or absence of events occurring with small probabilities, or any temporal logic can be used as verification properties [19,23,34]. When all possible temperature and humidity control tactics are verified (evaluated), the optimal one is selected.

#### 5.6 Executing Adaptation

***Principle.*** (Step 7) The chosen optimal adaptation tactic is applied to the managed system by the actuators of the system.

***Example.*** The adaptive air control system operates the selected optimal temperature and humidity control. The controls affect the indoor conditions through the system's actuators.

#### 5.7 PASTA Implementation

We also provide a PASTA reference architecture in Fig. 4 for the implementation of this approach. It is a layered architecture of an SAS with the PASTA approach. In the interaction layer, PASTA monitors the environment and managed system through the sensor and affects them through the actuators, like typical SASs. In the data analysis layer, there is a forecasting engine for the prediction of environmental changes and a knowledge management module for keeping the knowledge of the system up-to-date at all times. In the adaptation planner layer, a module searches for the optimal adaptation tactic through interactions with the adaptation verification layer. In the adaptation verification layer, the SMC module verifies an adaptation tactic governing the sample generator, the simulator, and the verifier.

The sample generator produces samples of the future environment based on the prediction of the forecasting engine. The simulator simulates the system model with an adaptation tactic in the given sample future environment. The verifier analyzes the simulation results to check the adaptation goal achievement, such as quality of service or invariant properties. In the knowledge layer, there is an environment database, a system model manager, an adaptation tactic repository, and an adaptation goal manager. This layer interacts with the others, providing and updating the knowledge of the SAS. This architecture is a reference, so it includes the essential components of an SAS with the PASTA approach and can be extended.

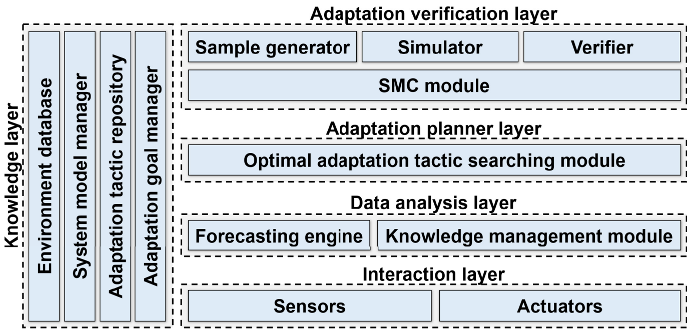

**Fig. 4.** PASTA reference architecture

In addition, to support engineers who develop SASs based on the PASTA approach, which was explained in the previous sections, we implemented a PASTA skeleton based on the reference architecture with guiding comments and released the source code on an open-source repository[1]. The skeleton is available in Java and Python. Engineers should write application-specific codes following comments tagged with "*todo*". The class diagram of the skeleton is presented in Fig. 5. An adaptation is activated by the "*adaptManagedSystem*" operator. It promotes easier PASTA implementation, allowing for the utilization of third-party libraries or tools for some components, such as the forecasting engine or the SMC module.

## 6 Evaluation

### 6.1 Research Questions

We demonstrate the feasibility of applying the PASTA approach as one efficient alternative to PMC-based proactive adaptation to SAS development. There are three research questions addressed.

***RQ1: (Cost efficiency of PASTA) How fast is PASTA's adaptation planning?*** PASTA leverages SMC for efficient adaptation verification at runtime. Although almost all existing proactive adaptation approaches utilize PMC for the runtime verification of adaptation tactics, the PASTA approach is one of the most efficient alternatives to PMC-based proactive adaptation approaches. To determine the efficiency of PASTA, we compare the application planning time of PASTA and the PMC-based adaptation. We confirm the differences in time consumption between SMC- and PMC-based approaches in solving proactive adaptation problems of the same complexities.

***RQ2: (Adaptation planning accuracy of PASTA) How accurately does PASTA search for the optimal adaptation tactic?*** PMC formally examines a probabilistic model and verifies whether it satisfies the given properties; however, SMC examines the given model with numerous sample simulation

[1] https://github.com/yongjunshin/PASTA

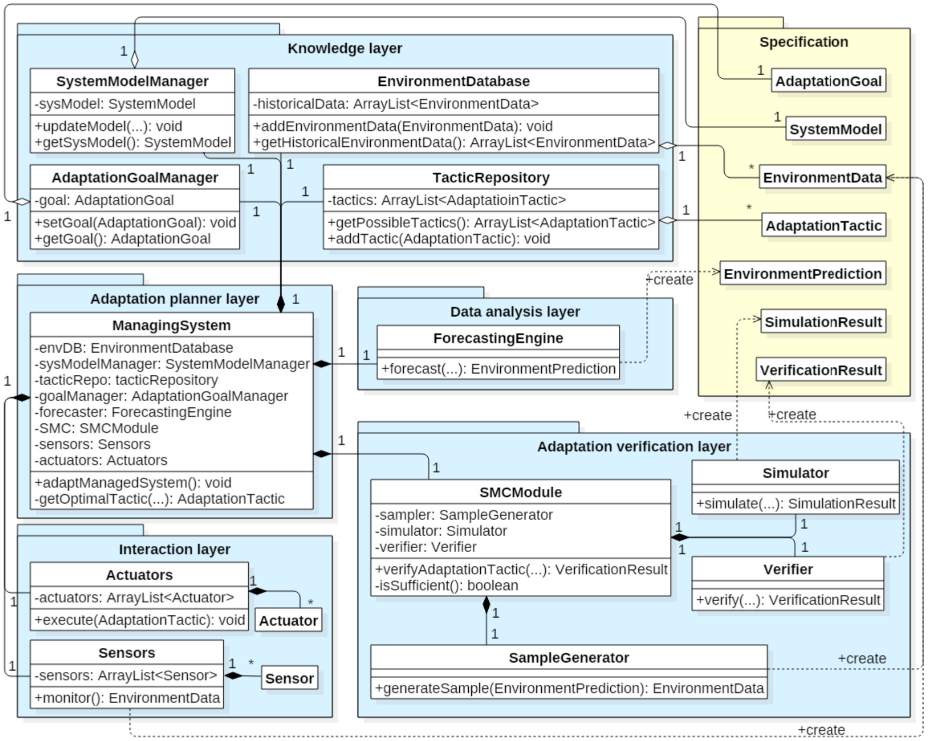

**Fig. 5.** Class diagram of the PASTA skeleton results, so it returns the statistical evidence of the model's properties and thus has the inevitable limitation that it can return inaccurate verification results limited to the finite number of samples. It is known that SMC can produce results similar to PMC [19,23,34], and for this research question, we compare the similar proactive adaptation planning results of PASTA with the planning results of the PMC-based approach. We determine how much accuracy has been lost by the cost savings identified in RQ1 as well as whether the loss of accuracy is acceptable.

***RQ3: (Adaptation performance of PASTA) How effective is the adaptation goal achievement performance of PASTA?*** For research question 3, we examine whether the PASTA approach is actually effective in achieving the adaptation goals of SASs. To evaluate the adaptation performance of PASTA, we compare the simulation execution results of approaches taking no adaptation, reactive adaptation, PMC-based proactive adaptation, and PASTA.

### 6.2 Evaluation Setup

We evaluate the PASTA approach using two example SASs. One is the adaptive air condition control system, the illustrative example of this paper, and the

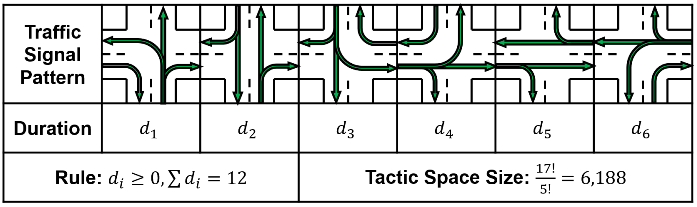

**Fig. 6.** Adaptation tactic of traffic signal controller other is an adaptive traffic signal controller of an intersection. The flow of cars in cities changes with the passage of time, which causes traffic congestion. A smart traffic signal controller that automatically controls traffic flow is a good example of applying proactive adaptation because changes in traffic conditions can be predicted based on historical data. Our signal controller predicts the traffic volume in an intersection and identifies an optimal configuration of signal patterns that minimizes the number of waiting vehicles. An actual signal controller is abstracted, and durations of signal patterns are dynamically controlled, as shown in Fig. 6. We applied PASTA to the two cases of different complexities and simulated them based on actual data acquired from public data repositories to make them realistic. Detailed descriptions of the two SASs and the evaluation setup are provided in Table 1.

We compared the adaptation cost, accuracy, and performance of the PASTA approach with the PMC-based proactive adaptation approach. The PMC-based proactive adaptation approach was implemented following a pioneering paper [26]. PRISM, a widely used probabilistic model checker, was utilized in the implementation [21]. We used default hybrid computation engine. The models of environments, systems, and tactics were specified in Markov decision processes (MDPs), and the adaptation goals were specified in the reward-based properties of the MDPs. As in paper [26], the following environmental changes have been predicted based on the data, and the PRISM modules have been constructed and verified based on the prediction. Thus, the optimal adaptation tactic has been found. In addition to the PMC-based approach, non-adaption and reactive adaptation approaches were also compared in terms of a system's goal achievement. For the PASTA approach, SMCS, the naivest SMC algorithm as explained in the background section, was implemented and evaluated by varying the number of samples used for the verification from 10 to 10000 (10, 100, 1000, 2000, ..., 9000, 10000).

#### 6.3 Evaluation Results

**RQ1:** We measured and compared the time spent on adaptation planning for both case systems using the PASTA and PMC-based approaches. The adaptation planning time includes modeling or sampling time and probabilistic or statistical verification time to identify the optimal tactic. Figs. 7 and 8 show the

**Table 1.** SASs for evaluation

| | **Adaptive air condition control system** | **Adaptive traffic signal controller** |
|---|---|---|
| **Environment** | Temperature and humidity condition | Car inflow to an intersection |
| **Environment complexity** | 2 environmental factors (temperature, humidity) | 12 environmental factors (the number of car inflow from 4 source roads to other 3 destination roads: 12 directions) |
| **Source of real environmental data** | Open weather data portal of - South Korea (https://data.kma.go.kr) - 2018 hourly weather data of Seoul | Open traffic data of Daegu, South Korea (https://car.daegu.go.kr) - 2018 Daily&Hourly Traffic data of an intersection in Daegu |
| **System** | Indoor air condition controller | Traffic signal controller |
| **System model** | Model of changing indoor temperature and humidity affected by environment conditions and the system's control | Model of changing the number of waiting cars in the intersection affected by car inflow and traffic signals |
| **Sensors** | Temperature sensor, humidity sensor | Traffic flow sensors for each 12 directions |
| **Actuators** | Temperature control actuator, humidity control actuator | Traffic lights |
| **Adaptation tactic** | Temperature control value, humidity control value | Configuration of traffic signal pattern duration |
| **Size of the adaptation tactic space** | 101 possible control values for each temperature and humidity by the system capability (-5, -4.9, ... +4.9, +5 ($°C$, %)) | 6,188 possible configurations of traffic signal pattern duration (Fig. 6) |
| **Adaptation cycle** | 1 hour | 1 hour |
| **Adaptation goal** | Target air condition (25, 50) - following ASHRAE comfort zone [8] | Minimizing the number of waiting cars |
| **Tactic evaluation criteria** | Average difference between controlled indoor condition and target condition | Average of the number of waiting cars |
| **Forecasting method** | Random walk model with seasonal differencing [30] | Polynomial regression |

evaluation results for each system. The reported planning time is the average of 100 repeated experiments. The adaptation planning time for the PMC-based approach is constant, but the time for PASTA increases in proportion to the number of samples used for the SMC because the time for a single simulation is almost constant. Unfortunately, the traffic signal controller was not able to obtain adaptation planning results using PMC with a 2G memory because its models and tactics were more complex than the air condition control system so consume larger verification resource. Therefore, for the traffic signal controller,

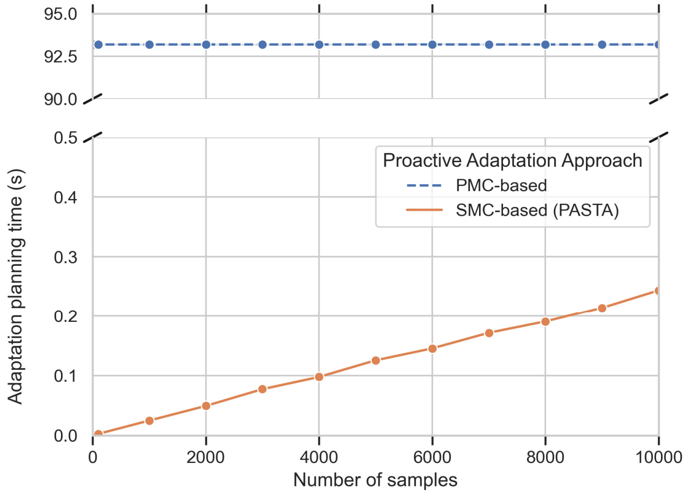

**Fig. 7.** Adaptation planning cost - Air condition control system

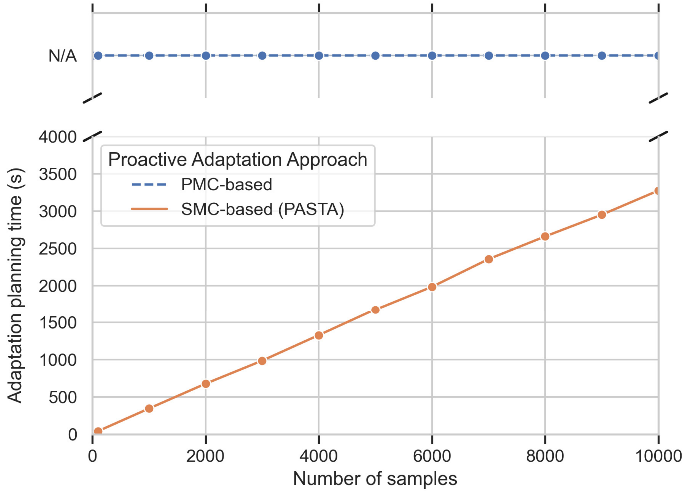

**Fig. 8.** Adaptation planning cost - Traffic signal controller the adaptation planning time for the PMC-based approach was not assigned; however, both systems confirmed that PASTA would complete adaptation planning much faster than the PMC-based approach. It was also confirmed that the adaptation planning time of PASTA is proportional to the number of samples and the complexity of the adaptation problem.

**RQ2:** To confirm the similarity of the optimal tactics that the PASTA and PMC-based approaches found, we compared the optimal tactics returned by the PASTA and PMC-based approaches in the same situation. To quantify the similarity, we defined two criteria. If the two tactics were the same, they were defined as *identical*, and if they were adjacent in terms of the tactic specifications, they were defined as *similar*. For example, for the air condition control system, temperature control tactics $+3°C$ and $+3.1°C$ were adjacent because the temperature control unit is 0.1C based on the system's capability, and the probability that arbitrarily two tactics are adjacent is less than 2%. Because the samples used by SMC are randomly generated, we repeated the PASTA experiments 100 times and report the percentage of identical or similar tactics compared to the tactic returned by the PMC-based approach. Because the traffic signal controller could not find the optimal tactic utilizing PMC, only the experimental results of the air condition controller are shown in Fig. 9. We could see that PASTA always found the same or similar optimal tactic as the PMC-based approach except when using 10 samples; however, one limitation of utilizing SMC is that regardless of how many samples we increased, we could not always obtain the same results as the PMC-based approach's results, which is considered an oracle. This case system returned accurate results at approximately 50% on average.

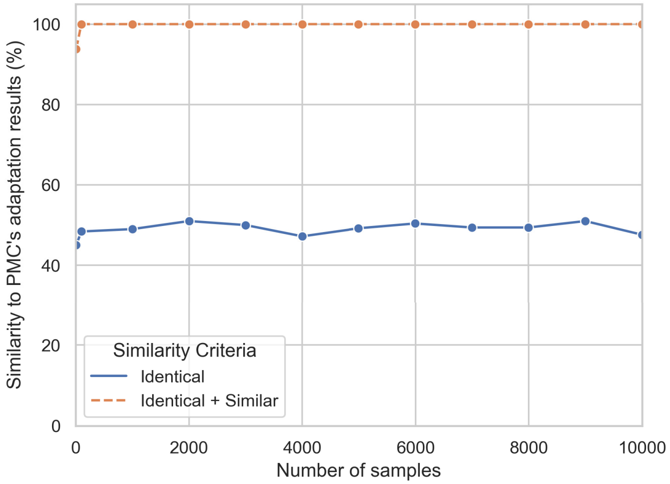

**Fig. 9.** Adaptation planning accuracy - Air condition control system

**RQ3:** For RQ1 and RQ2, we showed that PASTA can quickly find a sub-optimal adaptation tactic that is similar to the PMC-based approach's result. For RQ3, we obtained simulation results to confirm the adaptation performance of the PASTA approach in comparison with non-adaptation, reactive adaptation, and PMC-based proactive adaptation. As shown in Fig. 10, the goal of the air condition control system was to keep the temperature at $25°C$, and proactive adaptation approaches showed a better adaptation performance than other strategies. In addition, the PASTA and PMC-based approaches exhibited a similar performance because PASTA has always made similar adaptation decisions to

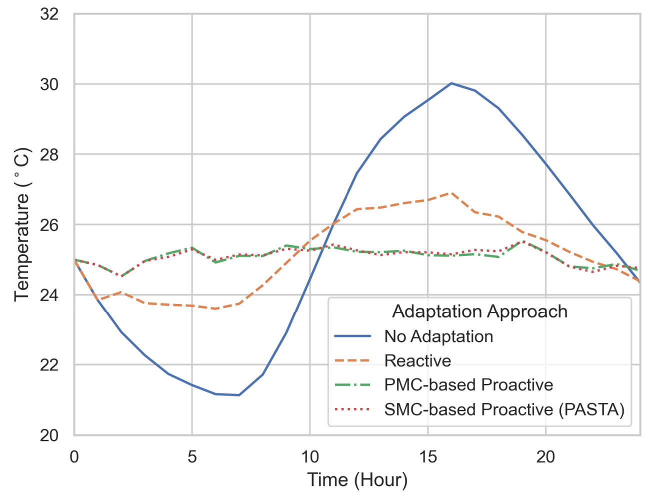

**Fig. 10.** Adaptation performance - Air condition control system

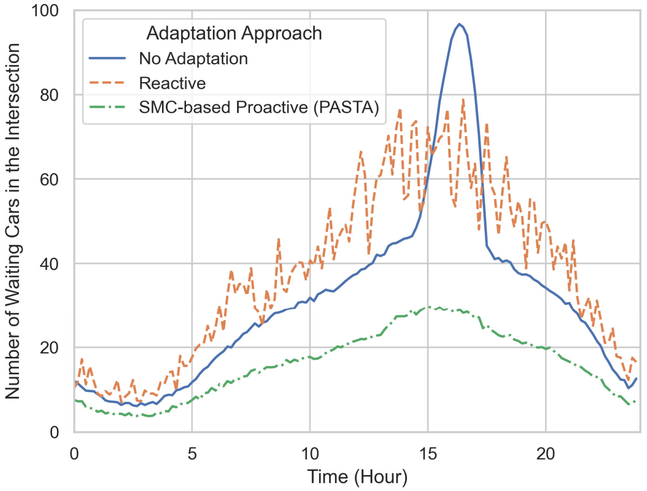

**Fig. 11.** Adaptation performance - Traffic signal controller the PMC-based approach. In Fig. 11, the goal of the traffic signal controller was to reduce the number of vehicles waiting at the intersection as much as possible, and proactive adaptation using PASTA showed the best performance. These two results demonstrate that proactive adaptation outperforms reactive adaptation and PASTA shows similar adaptation performance to the PMC-based approach with smaller verification cost.

**Table 2.** Comparison of proactive adaptation approaches

| | **PMC-based approach** | **SMC-based approach (PASTA)** |
|---|---|---|
| **Adaptation cost** | Forecasting time | Forecasting time |
| | Modeling time (relatively high) | Sampling time (relatively low) |
| | Probabilistic verification time (relatively high) | Statistical verification (Simulation + hypothesis testing) time (relatively low) |
| **Adaptation accuracy and performance** | Regarded as an oracle (high, limited to the quality of the models) | Provides similar adaptation results to PMC-based adaptation (relatively low, limited to the quality of the samples and models) |
| **Pros** | The optimal adaptation tactic can be found. | A sub-optimal adaptation tactic can be found with a lower adaptation cost. If the model can be simulated, it is not limited to a particular modeling language. |
| **Cons** | High adaptation cost is required. Modelling language is dependent on the model checker. | The adaptation result is not fully trustworthy. |
| **Proper application** | Safety-critical system | Real-time system |

We compared two approaches of proactive adaptation: PMC-based and SMC-based (PASTA) approaches. As we confirmed in our evaluation, the two approaches have their own advantages and disadvantages, so engineers should carefully decide which to choose for their SAS development. We summarized our insights regarding their characteristics in Table 2 to guide engineers' decision making. As we emphasized, the SMC-based approach makes adaptation decisions, verifying a system's adaptation tactics faster than the PMC-based approach. In addition, if it is possible to generate simulation results from the given models, the modeling language is not limited to the model checker; however, it is indubitable that an adaptation decision made by the SMC-based approach may not be globally optimal. Therefore, the SMC-based approach may not be suitable for some safety-critical systems, and the PMC-based approach could be the better choice if the trustworthiness of the system is the most important concern. For SASs requiring a lower adaptation cost, such as real-time systems, PASTA is more appropriate than the PMC-based approach.

### 7 Threats to Validity

One threat is the selection of the SMC algorithm. We selected SMCS to demonstrate the adaptation performance when selecting the simplest SMC algorithm. SMCS is suitable for explicitly indicating SMC-based adaptation costs affected by the number of samples, and all other SMC algorithms have similar characteristics. To reduce this threat, we also implemented SSP and SPRT and compared them to the PMC-based approach, and both showed similar cost, accuracy, and performance differences. Therefore, for this paper, only SMCS was selected and explained by varying the number of samples.

Another threat is the implementation of the PMC-based adaptation approach. We implemented the PMC-based approach directly following paper [26]. This threat was reduced because the authors published all the structures and codes of the PRISM module for the implementation of the approach. We implemented two case systems according to the PRISM module code shown in the paper. For a fair comparison, environment, system, and adaptation tactic spaces of the same complexities were given to both the PMC-based and PASTA approach.

### 8 Conclusion

We have proposed PASTA, a proactive adaptation approach using SMC, that is one efficient alternative to PMC-based proactive adaptation. We applied the PASTA approach to two realistic SASs. Through experiments based on actual data, we confirmed that PASTA would make an adaptation decision similar to the PMC-based proactive application approach in a shorter time. We then confirmed that the adaptation decision is more effective in achieving the system's goals than non-adaptation, reactive adaptation, and the PMC-based approach. Currently, PMC-based approaches are considered the major trend in proactive adaptation, but in this paper, we showed that the SMC-based proactive adaptation approach can be an efficient alternative. In addition, the algorithmic processes, reference architecture, and open-source skeleton of PASTA proposed in this paper will be of substantial help to developers who wish to apply PASTA to SAS development. This study was primarily conducted to validate the PASTA approach, but in the future, we plan to study methods such as effective sampling and adaptation space reduction for a more effective PASTA approach, and we also plan to apply PASTA to actual running systems.

### Acknowledgement

This research is partly supported by the MSIT(Ministry of Science and ICT), Korea, under the ITRC(Information Technology Research Center) support program (IITP-2020-2020-0-01795) supervised by the IITP(Institute of Information & Communications Technology Planning & Evaluation). This research is partly supported by IITP grant funded by MSIT (No. 2015-0-00250, (SW StarLab) Software R&D for Model-based Analysis and Verification of Higher-order Large Complex System). This research is partly supported by Next-Generation Information Computing Development Program through the National Research Foundation of Korea(NRF) funded by MSIT (2017M3C4A7066212).
